# Oligohexamethylene Guanidine Derivative as a Means to Prevent Biological Fouling of a Polymer-Based Composite Optical Oxygen Sensor

**DOI:** 10.3390/polym15234508

**Published:** 2023-11-23

**Authors:** Maxim D. Lisowski, Elizaveta V. Korobova, Alina O. Naumova, Igor P. Sedishev, Alina A. Markova, Minh Tuan Nguyen, Vladimir A. Kuzmin, Artemiy I. Nichugovskiy, Vyacheslav A. Arlyapov, Nikolay A. Yashtulov, Pavel V. Melnikov

**Affiliations:** 1 M.V. Lomonosov Institute of Fine Chemical Technologies, MIREA—Russian Technological University, 119571 Moscow, Russia; lisowskij.m.d@gmail.com (M.D.L.); korobova.nnov@gmail.com (E.V.K.); naumova_a@mirea.ru (A.O.N.); sedipa@list.ru (I.P.S.); nichugovskij@mirea.ru (A.I.N.); yashtulov@mirea.ru (N.A.Y.); 2 Emanuel Institute of Biochemical Physics, Russian Academy of Sciences, 4 Kosygin Street, 119334 Moscow, Russia; alenmark25@gmail.com (A.A.M.); tuantonyx@yahoo.com (M.T.N.); vak@sky.chph.ras.ru (V.A.K.); 3 Institute of Cyber Intelligence Systems, National Research Nuclear University MEPhI, 115409 Moscow, Russia; 4 Research Center “BioChemTech”, Tula State University, 92 Lenin Avenue, 300012 Tula, Russia; v.a.arlyapov@tsu.tula.ru

**Keywords:** oxygen sensor, optical sensor, antimicrobial properties, oligohexamethyleneguanidine, nanostructured surface, surface modification, adhesion control, fluorinated material, biofouling

## Abstract

The use of biocidal agents is a common practice for protection against biofouling in biomass-rich environments. In this paper, oligohexamethyleneguanidine (OHMG) polymer, known for its biocidal properties, was further modified with para-aminosalicylic acid (PAS) to enhance its properties against microorganisms coated with a lipid membrane. The structure of the product was confirmed by ^1^H NMR, ^13^C NMR, and FTIR spectroscopy. The values of the minimum inhibitory concentration (MIC) against *Mycobacterium smegmatis* ATCC 607 and *Pseudomonas chlororaphis* 449 were found to be 1.40 and 1.05 μg/mL, respectively. The synthesized substance was used as an additive to the polymer matrix of the composite optical oxygen sensor material. A series of samples with different contents of OHMG-PAS was prepared using a co-dissolution method implying the fabrication of a coating from a solution containing both polymers. It turned out that the mutual influence of the components significantly affects the distribution of the indicator in the matrix, surface morphology, and contact angle. The optimal polymer content turned out to be wt.3%, at which point the water contact angle reaches almost 122°, and the fouling rate decreases by almost five times, which is confirmed by both the respiratory MTT assay and confocal microscopy with staining. This opens up prospects for creating stable and biofouling-resistant sensor elements for use in air tanks or seawater.

## 1. Introduction

Modern human activity, including agro- and aquaculture, as well as various industries, is impossible without measuring and controlling the composition of various technological media, raw materials, and products. For this reason, the improvement of analytical systems is a necessary ongoing task, related in particular to the development of smart materials that can change or adjust their characteristics in response to influences from external sources [1]. A significant share of measured factors is occupied by physiologically important analytes, such as O_2_, CO_2_, and pH in biological systems, industrial facilities, and environmental objects [2,3]. Moreover, methods for measuring these parameters are used as transducers in biosensors, which are widely used as economical and fast analytical methods in situ [4,5,6,7]. A related but different area is measurement in media with a high biomass content, such as seawater, aerotanks, and bioreactors [8,9,10]. In such an environment, gradual biological fouling of the surface of both the sensor as a whole and the sensitive coating is inevitable, which leads to distortions in the readings [11].

To date, two types of technology have been widely used as transducers in sensory and biosensor systems: electrochemical and optical sensors [12]. Each has both advantages and disadvantages, which determine the applicability in a particular area [13]. For example, both electrochemical (Clark electrode) and optical (quenching of the phosphorescence of the indicator molecule) sensors can be used to measure the oxygen concentration [14]. Known disadvantages of the Clarke electrode are the consumption of O_2_ during measurement due to the reaction on the working electrode, the interfering action of a number of substances, such as H_2_S, a more time-consuming maintenance procedure, the drift of readings over time, and their dependence on hydrodynamic conditions. However, the method is well established, the sensor is easy to sterilize, and it is mechanically more reliable than fiberoptic sensors. Optical sensors are more expensive than electrodes, and a gradual photobleaching of the indicator occurs during operation; however, the interfering effects are much less pronounced. In particular, there is no effect of hydrogen sulfide.

A comparison of the two methods under real measurement conditions showed the advantage of the optical sensor, especially in areas where customization of the sensor material is necessary [13,15,16]. In addition, the optical method shows resistance to poisoning under operating conditions in an environment with a high biomass content [17,18]. Various approaches are known for protecting the sensor matrix from biofouling. One of the methods is the use of fluorinated polymers [19,20,21], which have poor surface wettability and, accordingly, prevent the biomaterial from sticking. However, in this case, the problem with the solubility of the indicator may arise, in addition to the non-linearity of the calibration dependence, which is sometimes very significant [22]. Modified mesoporous materials obtained by the sol–gel process also have physical protection against particle adhesion [2,19,23,24]. In this case, both fluorine-containing substituents, which reduce the wettability of the surface [25] and bactericidal coatings can be used, for example, phosphorylcholine groups [23]. However, the presence of micropores can lead to contamination over time, and exposure to solvents can lead to the indicator dye leaching.

The creation of polymer-based composites makes it possible to some extent to overcome the disadvantages of the methods described above [2,26]. The sensor surface remains non-porous, and the calibration dependence is linear in the Stern–Volmer coordinates due to the presence of the mesoporous phase [20,27]. Moreover, fine tuning of the surface wettability is possible, both in the direction of improving the adhesion of the biomaterial to create a biosensor, and in the direction of imparting bactericidal properties [28]. However, the bactericidal action of functional groups attached to the surface is limited, and the introduction of releasable components may be preferable for a more prolonged action.

One of the ways to modify the properties of an optical sensor is the blending of different polymers in matrix and composition optimization [29]. The introduction of a biocidal component of a wide spectrum of action into a sensitive membrane could make it possible to prevent biofouling and make the sensor easier to use in media with a high biomass content. In this work, we used a cationic polyelectrolyte based on branched oligohexamethyleneguanidine (OHMG). OHMG derivatives are readily available [30,31], they have prolonged biocidal properties, and they are highly effective against a wide range of bacteria, viruses, and fungi, while showing low toxicity to multicellular organisms, in particular humans [32,33]. Modification of OHMG with para-aminosalicylic acid (PAS) or isopropylglycidyl ether eliminates one of the disadvantages of OHMG, namely low activity against pathogens which have a lipid shell [34]; therefore, this particular type of biocidal polymer was used in this work. The conditions for introducing the OHMG derivative into the polymer matrix of the composite optical molecular oxygen sensor were optimized, and the antibacterial effect of the resulting coatings was tested.

## 2. Materials and Methods

### 2.1. Materials

A sample of branched OHMG hydrochloride (“DEZAPOL^®^”) was obtained from Institute of Pharmaceutical Technologies (JSC “Institute of Pharmaceutical Technologies”, Moscow, Russia), the number-average OHMG molecular weight was 1103 Da, the average number of branches was 0.55 per molecule, and the average number of monomeric units n = 6 [33].

All other reagents and solvents used were chemically pure and produced by ChemMed (ChemMed, Moscow, Russia) and Sigma–Aldrich (Sigma-Aldrich, Saint Louis, MO, USA).

### 2.2. Synthesis of a Complex of Branched Oligohexamethyleneguanidine and Para-Aminosalicylic Acid

**Scheme 1:**
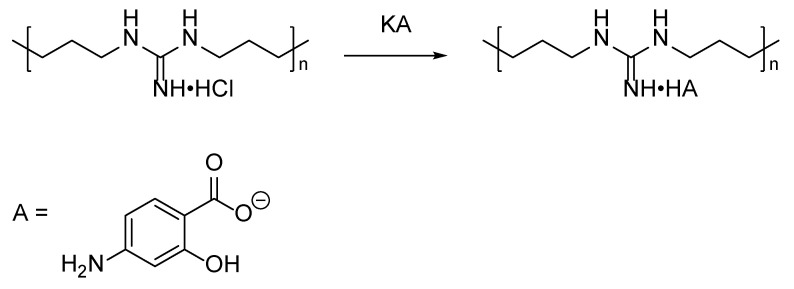
Reaction for obtaining the OHMG-PAS complex.

**Figure 1:**
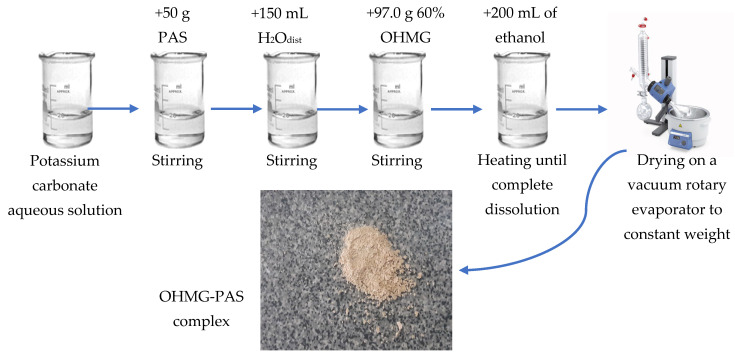
Scheme for obtaining the OHMG-PAS complex.

The reaction was carried out according to Scheme 1. The sequence of steps is shown in Figure 1. A total of 22.0 g of potassium carbonate was added to the beaker and 75 mL of distilled water was added followed by stirring until the complete dissolution of salt (approximately 10 min). Then, 50.0 g of PAS was gradually added to the solution over 40 min with constant stirring, which was accompanied by the release of carbon dioxide. The system was diluted with 150 mL of distilled water, which is necessary to dissolve the resulting salt. Then, 97.0 g of 60% solution of OHMG was added over 15 min while stirring at a medium speed. A total of 200 mL of ethanol was added to the system after precipitation of OHMG-PAS complex. The mixture was heated to boiling for about 10 min, followed by ethanol addition until the product was completely dissolved. After cooling for 24 h, the precipitated product was separated, washed, and dried on a vacuum rotary evaporator for about 1 h to constant weight. The weight of the pure product was 80.5 g; the yield was 85.0%. The total time spent on synthesis was 26 h.

### 2.3. OHMG-PAS Complex Characterization

#### 2.3.1. NMR Spectroscopy

The structure of the synthesized OHMG-PAS complex was confirmed by NMR spectroscopy. ^1^H NMR and ^13^C NMR spectra of OHMG-PAS solution in deuteromethanol were recorded on a Bruker DPX-300 NMR Fourier spectrometer with a superconducting magnet. The identification of the obtained substances was carried out by comparing the spectra of the sample with published reference spectra [35]. Quantitative determination of the composition of OHMG derivative was carried out by analyzing the integral signal intensities.

The chemical shifts in the signals are given relative to the internal standard (MeOH).

#### 2.3.2. Fourier Transform Infrared Spectroscopy (FTIR)

The presence of characteristic functional groups in the resulting polyelectrolyte was confirmed by the position of the characteristic bands in the FTIR spectrum. The spectrum was obtained by means of Spectrum 65 FT-IR spectrometer (Perkin Elmer, Waltham, MA, USA).

#### 2.3.3. Determination of Minimum Inhibitory Concentration (MIC)

The minimum inhibitory concentration is the lowest concentration of a chemical that prevents visible growth of bacteria or fungi in vitro. The study was conducted under sterile conditions. Bacterial reference strains used in this research were *Mycobacterium smegmatis* ATCC 607 and *Pseudomonas chlororaphis* 449. The source of the strains was the interclinical bacteriological laboratory of I.M. Sechenov First Moscow State Medical University (Moscow, Russia). This choice of microorganisms was due to the published data [36].

The sensitivity of bacteria was determined by the standard broth macrodilution method. Strains were tested in the cation-adjusted Mueller–Hinton broth (provided by State Research Center for Applied Biotechnology and Microbiology, Moscow region, Russia).

For the preparation of the bacterial suspensions, net daily cultures of microorganisms were diluted in sterile 0.9% sodium chloride solution until a turbidity equivalent to a 0.5 McFarland standard was reached (approximately 1.5 × 10^8^ CFU/mL). A total of 8 mL of broth and 100 µL of bacterial suspension were added to the prepared sterile Petri dish. Then, stirring was performed to obtain a homogeneous medium. Next, the 96-well culture plate was filled with the resulting mixture by means of the 8-channel pipette; 100 µL was added into each well. Then, 100 μL of a 2% OHMG-PAS solution in dimethyl sulfoxide (DMSO) was added to the first column of the plate. Twofold dilutions were carried out in further columns by subsequent transferring of 100 μL from the previous column with an eight-channel pipette. The last column was used as a growth control zone. The culture plate was incubated at the temperature 36 °C for 24 h. The detection of the MIC was estimated on the absence of growth in the medium containing the lowest concentration of the OHMG-PAS.

### 2.4. Sensor Fabrication

A previously developed composite material for an optical sensor of molecular oxygen was taken as the basis for modification [11]. It consists of two main phases: (i) SiO_2_ microparticles with an indicator dye distributed in the mesopores and (ii) a polymer base that serves as a medium for the distribution of particles and protects them from the interfering influence of the analyzed medium. Pt (II) 5,10,15,20-tetrakis (2,3,4,5,6-pentafluorophenyl)-porphyrin (PtTFPP, Frontier Scientific, Logan, UT, USA) and Merck silica gel 60 (Merck KGaA, Darmstadt, Germany) were used for the first phase, and fluorine-containing polymer fluoroplastic 42 (F42, HaloPolymer, Moscow, Russia) was used as a gas-permeable membrane base.

The co-dissolution method was chosen to introduce the OHMG-PAS into the sensor membrane surface, because it allowed us to achieve a uniform distribution of both components in the resulting material providing prolonged bactericidal action. First, two solutions were prepared: (I) polymer solution and (II) OHMG-PAS solution. Polymer solution was obtained by adding 7.5 mL of acetone to 0.6 g of F42, followed by stirring until a homogeneous medium was formed (about 10 min). OHMG-PAS solution was prepared by adding 2.5 mL of methanol and 0.5 mL of DMSO to 0.05 g of OHMG-PAS. The mixture was kept for 5 min on a magnetic stirrer until complete dissolution. Mixing the prepared solutions gave solution III, and varying the volume ratio made it possible to change the content of OHMG-PAS in the resulting material. The final volume of solution III was adjusted with a mixture of methanol and DMSO (5:1 ratio) so that the final ratio of solvents was the same in all solutions and the conditions for obtaining coatings were the same. The studied ratios of components are shown in Table 1. Solution III was left on the stirrer for 15 min until the mixture was completely homogenized.

Next, the fabrication of the composite sensor material was carried out similarly to the previously published procedure [11]. A total of 0.12 g of silica gel with the core–dye–shell structure was added to obtained solution III, and the mixture was placed in an ultrasonic bath for 20 min to allow even distribution throughout the volume of the solution. The resulting substance was dropped onto a glass slide using a 1 mL automatic pipette and distributed over the surface using a knife coating device with a 200 μm gap. The sample was dried in the dark for 24 h.

### 2.5. Sensor Characterization

#### 2.5.1. Characterization of the Sensor Performance

Key sensor characteristics, such as calibration, response time, and photostability were assessed according to procedures developed and described previously [11]. Calibration dependencies for the composite material were obtained using a homemade setup that allows one to set the pressure in the system in the range from atmospheric (P_atm_) down to 2 × 10^−3^ kPa (1.5 × 10^−2^ Torr), which was taken as an oxygen-free environment. The pressure measurement error was within 0.250 kPa (1.9 Torr).

The response time was determined at a constant temperature as the time interval during which the sensor readings reached the level of 95% (t_95_) of the final stationary value. The response time when the sensor was placed from an oxygen-free environment (water purged with an inert gas) into an air-saturated environment (t_1_) and the time of the reverse process (t_2_) were determined separately.

The photostability of the composite material was assessed by the decrease in phosphorescence intensity under continuous illumination by a matrix of LEDs with a maximum emission at a wavelength of 405 nm and a total power of 7 W.

#### 2.5.2. Determination of the Antimicrobial Activity

The well-known disc diffusion test was performed with the samples to determine the zone of growth inhibition [37]. The experiment was performed under sterile conditions. The bacterial suspensions net daily cultures of microorganisms (*Mycobacterium smegmatis* and *Pseudomonas chlororaphis*) were diluted in sterile 0.9% chloride solution sodium until a turbidity equivalent to a 0.5 McFarland standard was reached (approximately 1.5 × 10^8^ CFU/mL). A total of 1 mL of the suspension solution was added to the sterile Petri dishes filled with agar and stirred thoroughly. Four pieces with an area of 1 cm^2^ were taken from the sample and added to the dish so that the samples were at a large distance from each other. The agar plates were kept at 38 ℃ for 24 h, and then the size of the zone of growth inhibition was estimated.

#### 2.5.3. Water Contact Angle and Surface Free Energy

The water wetting angles θ of the samples were measured with an Easy Drop DSA100 instrument (KRUSS, Hamburg, Germany) and drop shape analysis software ADVANCE (version 1.14, KRUSS, Hamburg, Germany) with averaging over three points of the surface of each sample in four replicates for each OHMG-PAS concentration (the uncertainty of a single measurement ±1°). Calculations of surface free energy and its dispersive and polar components were carried out using the OWRK method by means of the instrument software. Water and diiodomethane were used as test liquids.

#### 2.5.4. The Morphology of the Surface

The surface morphology was assessed by two methods: scanning electron microscopy (SEM) and optical micrographs obtained by means of the digital microscope.

To obtain SEM images, the samples were fixed on an aluminum table with double-sided conductive carbon tape. A total of 20 nm of chromium and 10 nm of carbon were sequentially deposited onto the surface of the samples by means of Cressington 208HR and Cressington CARBON HR magnetron sputtering devices (Cressington Scientific Instruments, Watford, UK), respectively. The samples were studied using a field emission scanning electron microscope (Hitachi SU8000, Hitachi High-Technologies Corporation, Tokyo, Japan). Images were acquired using a secondary electron detector at an accelerating voltage of 1 kV.

Microphotographs of the samples of each concentration were taken with an area of 1 cm^2^ in the form of digital micrographs using an eScope Pro DP-M17 USB microscope (OiTEZ, Shatin, Hong Kong, China).

#### 2.5.5. Evaluation of Biofilm Formation

The rate of biofilm formation was estimated using the results of the respiratory MTT assay and confocal laser scanning microscopy (CLSM) during in vitro experiment. The samples in three repetitions were incubated for 3 h, 24 h, and 3, 5, and 7 days in growth medium with *Mycobacterium smegmatis* at 28 °C.

The colorimetric MTT assay was used for the estimation of the respiratory activity of the biofilm formed on the sensitive elements’ surfaces [38,39]. This method allows one to measure the viability of cells by assessing the ability to reduce the yellow salt of 3-(4,5-dimethylthiazol-2-yl)-2,5-diphenyltetrazolium bromide (MTT). After reaching the end of incubation time, the samples were subsequently removed from the thermostat and placed in 10 mL test tubes. A total of 3 mL of a 0.1% MTT was added to the test tube and left for 3.5 h at 29 °C. Then, the liquid was drained, and 3.5 mL of DMSO was added to the stained samples and left for 45 min to extract the dye from biofilms. The optical density of the extract was determined at a wavelength of 590 nm using an Expert-003 photometer (Econix-Expert, Moscow, Russia). The biofilm formation was judged by the color intensities of the obtained solutions (OD_590_ MTT), which were directly related to the number of viable microorganisms.

Biofilms were stained with 2 mg/mL rhodamine B (Thermo Fisher Scientific, Waltham, MA, USA) in 1X PBS by immersing the sample in the solution for 30 sec, then washing the sample to remove unbound dye three times with 1X PBS [40]. Confocal laser scanning microscopy (Leica SP5 microscope (Leica, Wetzlar, Germany)) made it possible to visualize the stained biofilm, and the rhodamine B signal was recorded in 1X PBS at Ex 576, Em 590–700 nm, analyzing 5 frames per sample. The Nomarski contrast method allowed for detecting undyed particles. An argon laser with a wavelength of 510 nm was used for dye excitation. The resulting images were analyzed using the LAS X (version 1.4.5, Leica, Wetzlar, Germany) and ImageJ 1.54e software package with the BioFormats 5.8.2 plugin.

## 3. Results and Discussion

### 3.1. OHMG-PAS Complex Characterization

**Figure 2:**
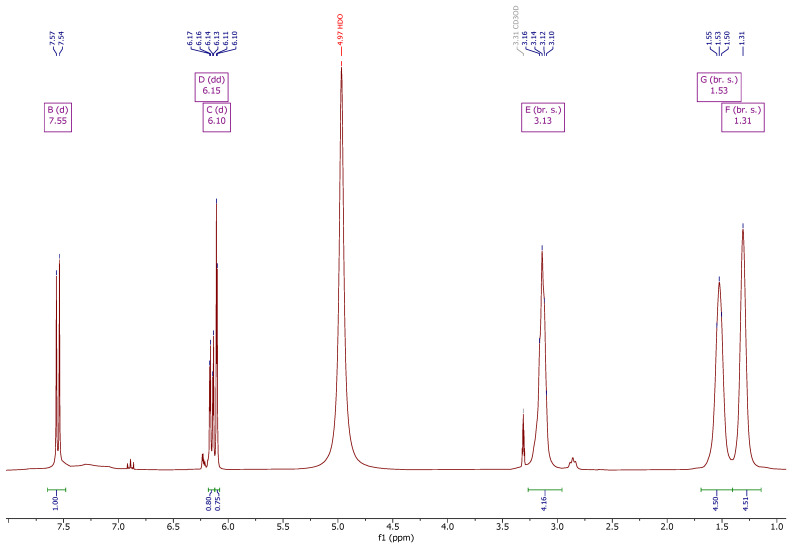
^1^H NMR (300 MHz, MeOH) of the OHMG-PAS complex.

During this investigation, we conducted NMR spectroscopy on deuteromethanol to elucidate its carbon skeleton structure. The ^1^H NMR spectrum (Figure 2) displayed prominent signals corresponding to OHMG groups in the chemical shift range of 1.1–3.1 ppm, along with signals appearing as doublets (with coupling constants J = 2.2 and J = 8.4 Hz) and one signal in the form of a doublet of doublets (with coupling constants J = 2.2 and J = 8.4 Hz) in the weak-field region. These spectral features are indicative of an AMX spin system, associated with a fragment of para-aminosalicylic acid present in the overall molecular structure.

**Figure 3:**
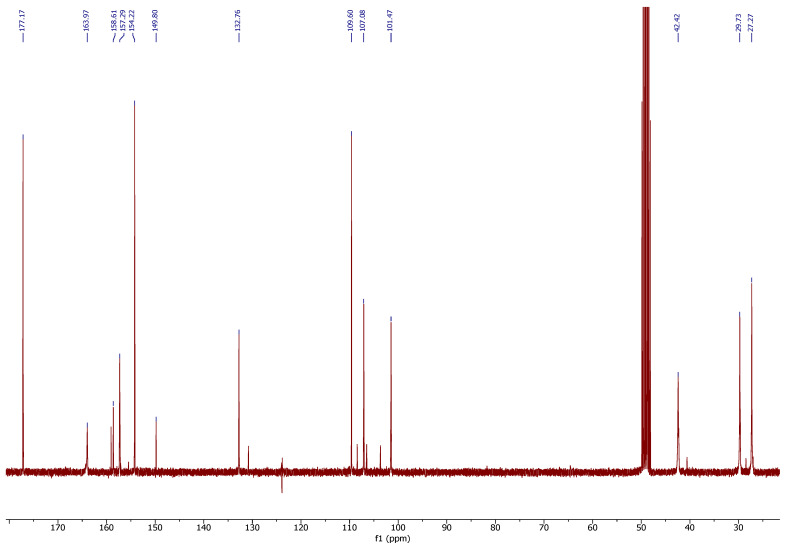
^13^C NMR (75 MHz, MeOD) of the OHMG-PAS complex.

Moreover, the ^13^C NMR spectrum (Figure 3) exhibited signals corresponding to the OHMG group and characteristic signals of the PAS fragment. The most intense signals (δ 27.3, 29.7, 42.4, 101.5, 107.1, 109.6, 132.8, 149.8, 154.2, 157.3, 158.6, 164.0, and 177.2) were attributed to carbon atoms within the main chain, suggesting a high concentration of this moiety within the molecule under investigation. Signals of lower intensity were observed for carbon atoms in terminal segments of the polymer chain and branched fragments, which corroborate the findings from prior studies [35]. These findings make a significant contribution to the comprehension of the carbon skeleton structure of the studied molecule and provide valuable data for further research in the domains of organic chemistry and molecular biology.

**Figure 4:**
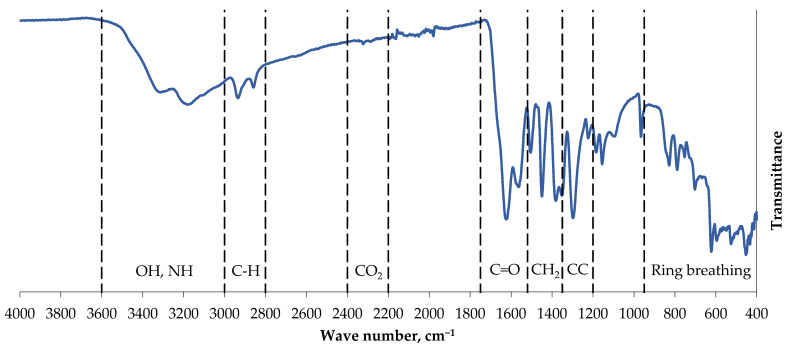
IR Fourier spectrum of OHMG-PAS complex.

The FTIR spectrum of the OHMG-PAS is shown in Figure 4. Wide overlapping peaks with absorption band maxima at 3200 and 3300 are due to =N-H and O-H stretching vibrations. Peaks corresponding to C−H asymmetric stretching of methyl and -CH_2_- groups (2930 cm^−1^), C-H symmetrical stretching of methyl, and -CH_2_-groups (2855 cm^−1^) are superimposed on them. The peak at 1460 cm^−1^ corresponds to -CH_2_- bending vibration. An intense double peak in the region of 1700–1520 cm^−1^ is characteristic of stretching vibrations in the C=O bond in carboxylic acids. The peak with an absorption maximum of 1300 cm^−1^ is characteristic of the CC bond stretching in the PAS molecular fragment. The region with overlapping peaks with wavenumbers less than 950 cm^−1^ is attributed to various modes in the aromatic ring of the PAS [41]. A small artifact is visible in the region of 2200–2400 cm^−1^, corresponding to CO_2_ present in the atmosphere. The results are consistent with the known spectra of other OHMG derivatives and PAS [41,42,43].

Antimicrobial activity is an important parameter for the evaluation of the effectiveness of the biocidal component in the matrix on the surrounding microorganisms. OHMG used as an initial substrate has proven bactericidal activity [30], but changing the structure of the molecule during modification can significantly alter its properties. Therefore, we used a standard test to determine the minimum inhibitory concentration (MIC) against *M. smegmatis* and *P. chlororaphis*. The measured values were 1.40 and 1.05 μg/mL, respectively. The results confirm that the synthesized complex indeed retained its own bactericidal activity, which is approximately at the level of other OHMG derivatives [36].

### 3.2. Sensor Characterization

Next, we proceeded to study the properties of the composite material of the optical oxygen sensor with OHMG-PAS embedded in the polymer matrix. First of all, we tested whether changing the composition affects such key properties as calibration dependence, response time, and photostability.

It turned out that the calibration dependence of the sensor material did not practically change compared to the material without the addition of OHMG-PAS. It remained linear over the entire range of oxygen partial pressures. This was expected, since the sensory properties of the composite material are due to the core–dye–shell microparticles, whose structure and properties did not change, since the polymer serves solely as a host matrix.

Sensor response time was estimated as the time needed to establish 95% of stable readings when the sensor was moved out of water with 100% oxygen saturation to an oxygen-free environment and back again. In the first case, it was 70 s, while for the backward process it was 60 s. For a gas environment, these characteristics were 8 and 6 s, respectively. These values are slightly higher than for pure F42 [11]. This may be caused by a decrease in the rate of oxygen diffusion in the polymer due to a decrease in the proportion of the fluorinated phase.

The phosphorescence intensity under constant irradiation decreases by 3% per day, and there is also no difference in this property with the material without the addition of OHMG-PAS. This was also expected, since the characteristic depends on the indicator used and the method of its encapsulation in the core–dye–shell structure, which did not change. However, if we evaluate the photostability of the entire material, the appearance of a yellow color of the polymer was observed after irradiation. The latter is apparently associated with the process of OHMG-PAS decomposition, which is not protected by fluorinated groups, unlike the polymer and the indicator molecule.

The measured diameters of microbial growth inhibition zones for various quantities of the biocidal component OHMG-PAS in the polymer matrix are presented in Table 2. It can be seen that the increase in the growth inhibition zone directly depends on the concentration of the biocidal component in the sample. It should be noted that there is a significant increase in antimicrobial activity when the complex content is more than 3%. The size of the growth inhibition zone also varies depending on the strain, and for *P. chlororaphis* the values obtained are noticeably larger, which correlates with the results obtained by the MIC method. A lower MIC value indicates that less substance is required for inhibition; therefore, under identical experimental conditions, a lower MIC value should correspond to a larger growth inhibition zone.

**Figure 5:**
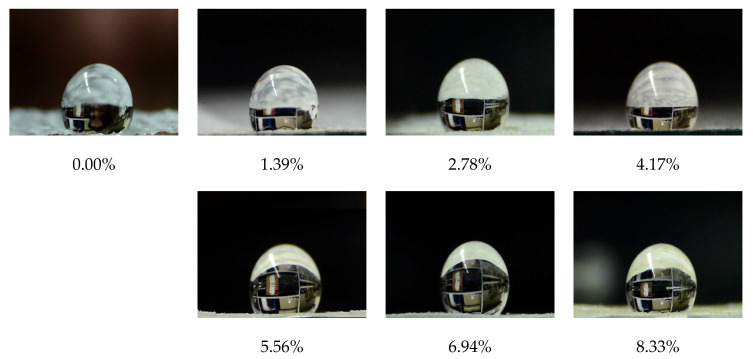
The influence of the OHMG-PAS concentration in the polymer matrix on the wetting of the sensor surface.

**Figure 6:**
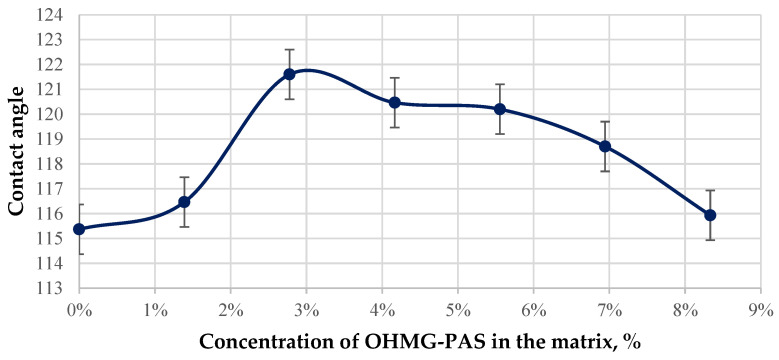
Dependence of the measured water contact angle on the concentration of OHMG-PAS in the polymer matrix of the sensor.

The effect of OHMG-PAS content on the wetting properties of the material is shown in Figure 5, and the dependence of the measured water contact angle on the concentration of OHMG-PAS is shown in Figure 6. An almost complete wetting of the surface is observed for a dispersive liquid, such as diiodomethane, and the contact angle is close to 0.

The dependence in Figure 6 is not linear and has a clear maximum in the concentration region of 2.8%, which indicates a more complex interaction of miscible polymer components, violating the law of additivity, which is typical for systems close to ideal. The values of surface free energy γ^tot^ and its dispersive γ^d^ and polar γ^p^ components were calculated using the OWRK method (Table 3). In general, the dependence mimics the graph in Figure 6, and a larger value of the contact angle corresponds to a smaller value of γ^tot^. It is clear when comparing the dispersive and polar components that the first dominates. This is expected, since fluorinated polymer F42 was used as the hosting matrix. The contribution of the polar component does not exceed 2% of the γ^tot^ value despite the presence of a polyelectrolyte in the structure of the material. The latter indicates that a change in the contact angle is most likely associated with a change in the morphology of the surface, and not with a change in its functional composition. The observed values and dependencies are consistent with the literature data for similar materials and polymer blends [44,45,46,47]. For example, for PVDF/PPEGMA blends, the contribution of γ^p^ becomes noticeable only when the PPEGMA content is more than 20 wt.%, while the contact angle drops already at a copolymer content of about 10 wt.% [44]. A slight increase in the contact angle is also observed at small amounts (about 3 wt.%), but Zhao et al. did not study the reasons for this phenomenon in detail.

**Figure 7:**
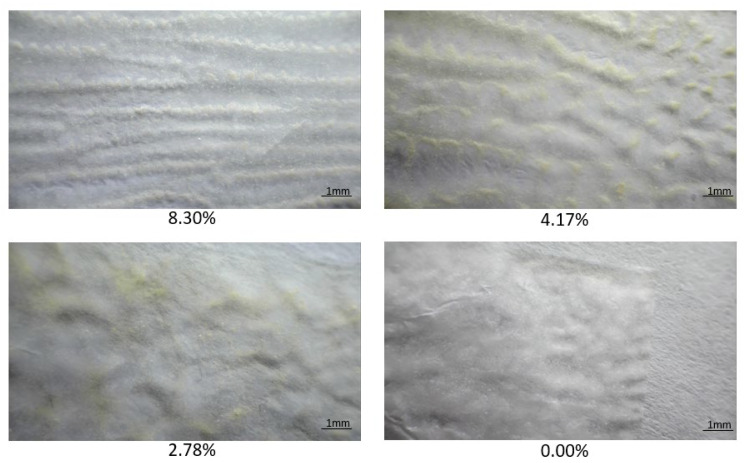
Micrographs of samples with different contents of OHMG-PAS.

The morphology of the samples was studied using SEM and optical micrographs to determine the origin of the observed dependence. Photomicrographs show that samples with different OHMG-PAS contents differ in structure and surface shape (Figure 7). It can be seen that SiO_2_ microparticles, which serve as carriers of the indicator dye, are distributed unevenly throughout the matrix at a high content of OHMG-PAS. A uniform distribution and the most homogeneous structures are observed for samples with OHMG-PAS concentrations of up to 3%; heterogeneous inclusions are visible throughout the material with concentration of 4.17%, and the inhomogeneities merge, forming parallel grooves at 8.30%. This violation of the homogeneity of the material leads to an increase in surface roughness at the microlevel, which apparently leads to deterioration in wetting. However, this heterogeneity does not affect the sensory properties of the material, because in the core–dye–shell system under consideration, they are provided by the structure of microparticles, which does not change [11].

**Figure 8:**
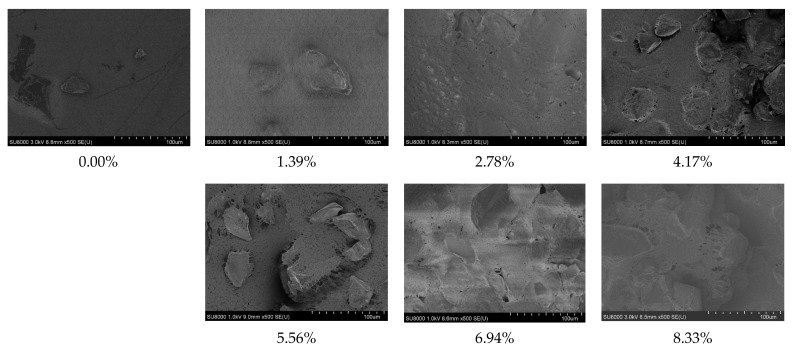
SEM images of samples with different contents of OHMG-PAS.

The reason for the observed phenomenon may be associated with the different polarities of the mixed polymers, which leads to the formation of the OHMG-PAS subphase with an increase in the content of the minor component. This hypothesis is confirmed by SEM images of the samples (Figure 8). It can be seen that the surface morphology remains virtually unchanged for OHMG-PAS concentrations less than 3%, although a small amount of inhomogeneity is observed for the 2.78% sample. The morphology changes significantly for samples with a high content of the bactericidal component. Uncovered grains of SiO_2_ are visible on the surface of samples with concentrations of 4.17% and 5.56%, which indicates a deterioration in adhesion. Micro-interfaces (boundaries separating areas of different brightness) between the phases formed by the two polymers become noticeable for samples with 6.94% and 8.33%.

In general, the morphology observations are consistent with the changes in the water contact angle as a function of OHMG-PAS content.

**Figure 9:**
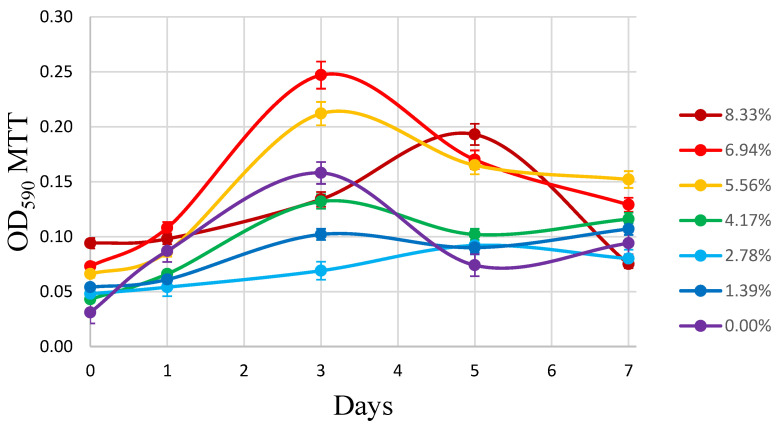
Change in respiratory activity of samples according to the results of the MTT assay (OD_590_ MTT) depending on the incubation time.

The change in respiratory activity associated with an increase in the number of living cells on the surface of the samples is shown in Figure 9. The dependences have the typical shape of a bacterial population growth curve but differ in amplitude and in the time of reaching maximum respiratory activity τ_max_. It can be seen that an increase in the content of OHMG-PAS in the matrix does not always lead to a lower rate of biofouling compared to unmodified material.

The fouling rate can be represented as the ratio of change in the OD_590_ MTT value to the time τ_max_ required to reach the maximum value, as follows:

R = (OD_590_ MTT_max_ − OD_590_ MTT_start_)/τ_max_. (1)

**Figure 10:**
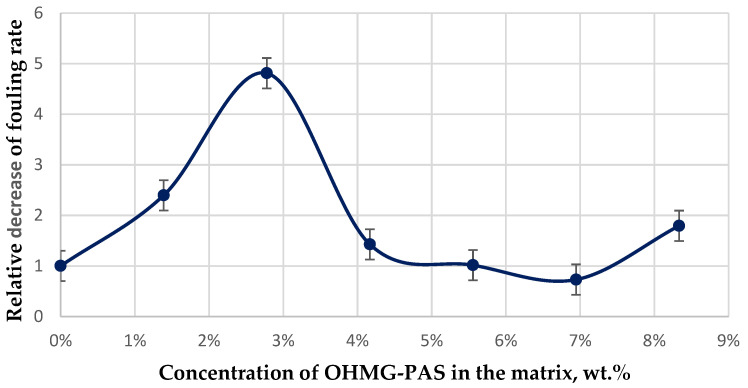
The dependence of the relative decrease in the fouling rate on the concentration of OHMG-PAS in the sample.

The calculated values are shown in Figure 10. The points are normalized relative to the fouling rate of unmodified material for ease of comparison. The dependence has a clear maximum in the region of 3%, exhibiting the decrease in the fouling rate by almost five times.

A decrease in the bactericidal action with a further increase in the content of OHMG-PAS may apparently be associated with a change in the structure of the surface of the material, confirmed by morphological analysis data above. It is also worth noting the matching of the maxima in Figure 10 with that observed in Figure 6, which allows us to make the assumption that biofouling is affected not only by the presence of a bactericidal polymer, but also by surface wettability. A similar effect was previously observed for sensors with modified nanodiamonds fixed in the surface [28], when an increase in the number of polar groups on the surface of the material did not lead to an increase in protective properties, but, on the contrary, promoted biofouling.

**Figure 11:**
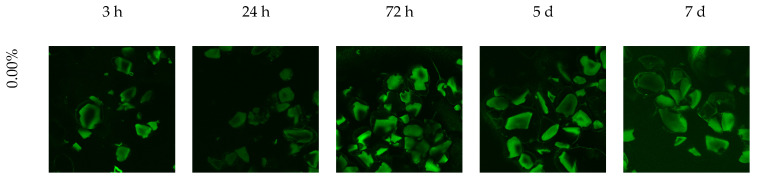

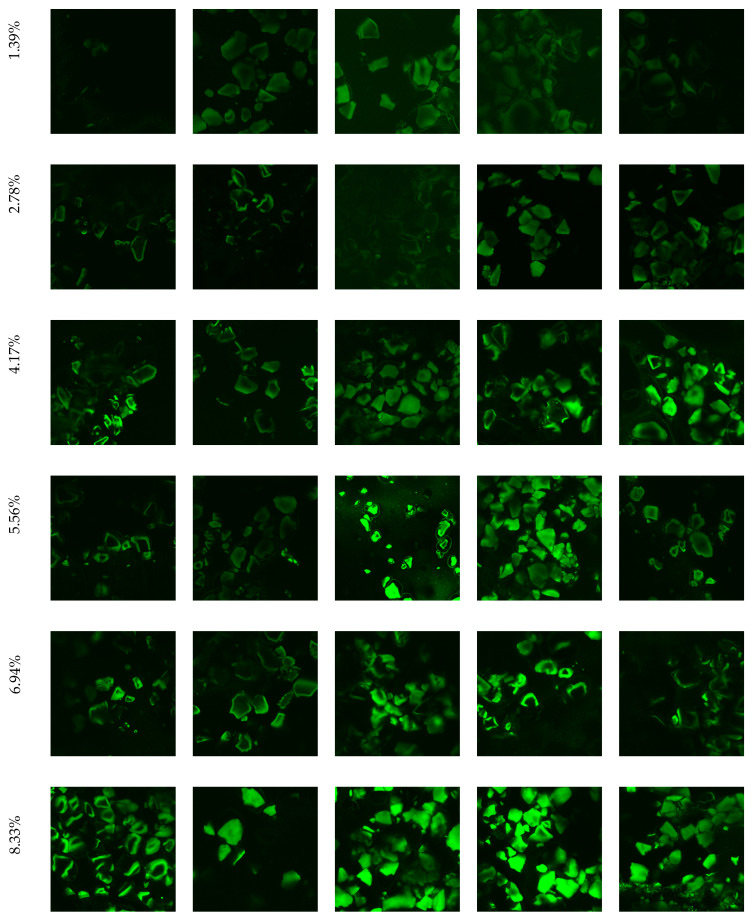
Images of modified polymer samples (1200×) after incubation in medium with *Mycobacterium smegmatis* at 28 °C for various times and fluorescent staining. The biofilms are shown in green. The concentration of OHMG-PAS in the samples is shown to the left of the images.

Confocal microscopy with staining was used to confirm the attachment of the biomaterial to the sensor surface (Figure 11). All images were obtained at a fixed excitation power, which makes it possible to estimate the number of microorganisms based on the fluorescence intensity in the green channel. The results of the treatment completely coincide with the data from the MTT assay. The lowest intensity is observed for the 2.78% sample, and we see almost the same and even greater fluorescence intensity for the 8.33% sample as for the sensor without the addition of OHMG-PAS. This confirms the hypothesis about the significant influence of the wettability of the material surface on the rate of fouling, even in the presence of the biocidal component.

## 4. Conclusions

The OHMG derivative with PAS was synthesized to increase its bactericidal activity against microorganisms with a lipid membrane. The structure of the resulting complex was confirmed by ^1^H-NMR, ^13^C-NMR, and FTIR spectroscopy. The bactericidal activity was tested against strains *Mycobacterium smegmatis* ATCC 607 and *Pseudomonas chlororaphis* 449. The values of the minimum inhibitory concentration (MIC) were found to be 1.40 and 1.05 μg/mL, respectively.

The possibility of using the synthesized compound as a biocidal additive to the material of a composite molecular oxygen sensor was investigated. It turned out that the nature of the interaction of the polymer components significantly affects the morphology of the sensor surface, its wettability, and the distribution of indicator dye carrier particles in the matrix. In the system under consideration, the law of additivity is clearly violated, and an increase in wetting of the material surface when the OHMG-PAS content exceeds 3% leads to a decrease in the protective properties. The observation was confirmed by a combination of physical and biological methods. Thus, the optimal content of the biocide complex was established, ensuring a reduction in the rate of biofouling by up to five times compared to unmodified material, which is undoubtedly important for operation in environments with a high biomass content, such as air tanks or seawater.

## Figures and Tables

**Table 1:** Ratios of a polymer solution (F42 in acetone), a solution of OHMG-PAS in a mixture of methanol + DMSO (ratio 5:1), and a mixture of methanol + DMSO (ratio 5:1) to obtain a series of samples with different contents of the bactericidal component in the polymer.

OHMG-PAS Content in the Polymer, wt.%	Polymer Solution, mL	OHMG-PAS Solution, mL	Methanol + DMSO (5:1 Ratio), mL
0.00%	15	0	6
1.39%	15	1	5
2.78%	15	2	4
4.17%	15	3	3
5.56%	15	4	2
6.94%	15	5	1
8.33%	15	6	0

**Table 2:** Measured values of growth inhibition zones for the samples.

OHMG-PAS Content in the Polymer, wt.%	Diameter of *M. smegmatis* Inhibition Zone, mm	Diameter of the *P. chlororaphis* Inhibition Zone, mm
0.00%	0.0	0.0
1.39%	0.8	1.0
2.78%	1.4	2.0
4.17%	4.2	6.7
5.56%	6.3	10.7
6.94%	7.3	13.3
8.33%	10.6	15.7

**Table 3:** Surface free energy of the samples with different OHMG-PAS contents.

OHMG-PAS Content in the Polymer, wt.%	γ^tot^_,_ mN/m	γ^d^, mN/m	γ^p^, mN/m
0.00%	18.08	17.80	0.28
1.39%	17.46	17.21	0.25
2.78%	14.54	14.45	0.09
4.17%	15.18	15.06	0.12
5.56%	15.34	15.21	0.13
6.94%	16.19	16.01	0.18
8.33%	17.76	17.50	0.26

## Data Availability

The data presented in this study are available on request from the corresponding author.
